# Ultrasonic-Responsive Pluronic P105/F127 Nanogels for Overcoming Multidrug Resistance in Cancer

**DOI:** 10.3390/gels11110878

**Published:** 2025-11-01

**Authors:** Shangpeng Liu, Min Sun, Zhen Fan

**Affiliations:** 1 Department of Polymeric Materials, School of Materials Science and Engineering, Tongji University, Shanghai 201804, China; shangpeng@tongji.edu.cn; 2 School of Materials Science and Engineering, East China University of Science and Technology, Shanghai 200237, China

**Keywords:** multidrug resistance, nanogels, ultrasound-responsive, drug delivery, enhanced retention effect

## Abstract

Effective management of multidrug-resistant cancers depends on effective, localized drug release and accumulation within the tumor microenvironment. In our work, Pluronic P105 and F127 mixed nanogels (PM) were fabricated through self-assembly to combat multidrug-resistant cancer. The approximate diameter of our prepared PM is 115.7 nm, an optimal size for tumor accumulation through the enhanced permeability and retention (EPR) effect. An in vitro drug release assay indicated that ultrasound could accelerate the drug release rate in doxorubicin-loaded Pluronic nanogels (PM/D). Additionally, the resistance reversion index (RRI) in the ultrasound-treated PM/D group was 4.55 and was two times higher than that in the free PM/D group, which represented better MDR reverse performance. Cell experiments demonstrated that, after 3 min of ultrasound, a greater amount of chemo-drug was released and absorbed by the MDR human breast cell line (MCF-7/ADR), resulting in significant cytotoxicity. Such enhanced therapeutic efficiency could be attributed to the combined effects of the two independent mechanisms: (i) ultrasound-controllable drug release realized effective release within resistant tumors with spatial and temporal precision and (ii) the contained Pluronic in the PM/D inhibited P-gp-mediated efflux activity to overcome MDR in tumors. Collectively, our findings support the feasibility of ultrasound-responsive PM as a drug-delivery platform for resistant cancers.

## 1. Introduction

In the past few years, the most important treatment for cancer has been chemotherapy [1]. However, multidrug resistance (MDR) is a major obstacle in the effective treatment of cancers, often leading to chemotherapy failure, cancer recurrence, and poor patient prognosis [2,3,4,5]. A key factor underlying MDR is the overexpression of efflux transporters such as P-glycoprotein (P-gp), which actively pump drugs out of cancer cells, thereby reducing their intracellular concentration to subtherapeutic levels [6,7]. Therefore, overcoming MDR in cancers requires two coordinated strategies: (i) inhibiting the efflux-dominated pharmacokinetics at the cell membrane and (ii) achieving deep intratumoural delivery with on-site, on-demand release to surmount the tissue-level barrier [8,9]. As many small-molecule drugs do not inherently satisfy these criteria, designing efficient drug-delivery systems that provide spatiotemporally controlled release and increase the effective intratumoural concentration is a promising and innovative approach.

Recently, extensive efforts have focused on developing stimuli-responsive drug-delivery systems to minimize the side effects of chemotherapy and enhance antitumor efficacy [10]. Nanotechnology has rapidly emerged in pharmaceutical sciences, providing capabilities that surpass conventional delivery strategies [11]. In particular, bioactive and externally responsive polymer materials have advanced tumor drug delivery through nanotechnology [12,13]. Rationally engineered nanoparticles can navigate the smallest capillary vessels and evade phagocyte capture to prolong blood residence. And nanoparticles can passively accumulate within tumors via the enhanced permeability and retention (EPR) effect. Encouragingly, a number of clinical studies show that nanoparticle-based formulations can substantially reduce the systemic toxicity associated with chemotherapy [14,15,16,17]. Among them, nanogels—hydrophilic, cross-linked polymer networks—are a highly attractive nanocarrier for reversing MDR. Their physicochemical properties can be tuned by varying polymer concentration, hydrophilic–hydrophobic balance, cross-linking density, and formulation conditions such as pH, ionic strength, and temperature [18,19,20]. Owing to their high water content and cross-linked polymer networks, nanogels are able to support high drug payloads and enable stimuli-responsive release (pH, enzymes, and temperature) to achieve on-demand spatiotemporally controlled release [21,22]. For example, Zhou et al. prepared a series of biocompatible core–shell nanogels for responsive drug release. The nanogels can stabilize curcumin from degradation at pH = 7.4 and release it in response to heat within the physiological temperature range [23]. Therefore, nanogels hold great promise as a versatile platform for overcoming cancer MDR.

Pluronics are composed of polyethylene glycol (PEG) and polypropylene glycol (PPG) and can be applied as an emulsifier, solubilizer, and permeation enhancer [24,25]. Notably, Pluronics were found to reverse MDR in cancer cells. Pluronics can insert into MDR cell membranes, perturb lipid microdomains, and reach mitochondria, inducing mitochondrial depolarization and inhibiting respiratory chain complexes to deplete ATP [26,27]. This depletion suppresses ATP-driven P-gp transport, thereby increasing intracellular drug retention. Concurrently, Pluronics directly inhibit P-gp ATPase and disrupt raft organization, further reducing efflux activity [28,29,30]. For example, Mahajan et al. used various Pluronics (Pluronics L61, P85, and F127) to prepare a series of pDNA-loaded formulations, which demonstrated good biocompatibility and low system toxicity and promoted gene expression [31]. Likewise, Meng et al. prepared Pluronic F127 micelles for targeted drug delivery with excellent biocompatibility and obvious therapeutic effects for MDR cancer [32]. Nevertheless, achieving rapid and precise on-site drug release at the tumor remains challenging. Stimuli-responsive designs, which are triggered by pH, ROS, enzymes, hypoxia, light, ultrasound, or heat, enable spatiotemporally controlled release at the disease site. Among these, ultrasound is a kind of sound wave with high energy that possesses good directionality, excellent penetrating ability, and controllable intensity [33,34,35]. Moreover, the energy of ultrasound can be transmitted to the chemical bonds within compounds and compound-involved materials to promote their movements [36,37,38]. Therefore, pairing ultrasound-triggered release with co-delivery of a chemotherapeutic and a P-gp inhibitor is particularly attractive. This synergy can effectively elevate intracellular drug levels beyond the efflux threshold, thereby combating MDR in cancer.

**Figure 1:**
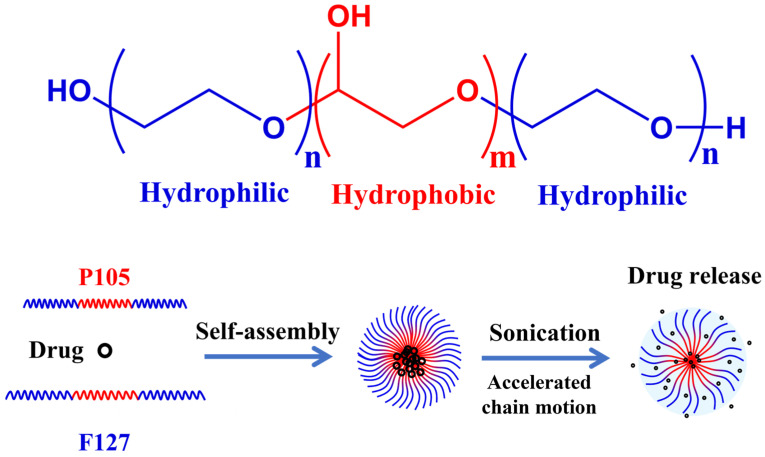
The chemical composition of the Pluronic polymer and the drug loading and ultrasound-controllable drug release process.

In this study, we developed self-assembled mixed nanogels composed of Pluronic P105 and F127 (PM) as a nanoscale delivery platform for combating MDR in cancer. As illustrated in Figure 1, the Pluronic P105 and F127 self-assembled into nanogels via hydrophilic and hydrophobic interactions, with a hydrophobic interior for drug sequestration and a hydrophilic exterior. We hypothesized that brief ultrasound would accelerate payload release from PM by perturbing nanogel packing, while Pluronic components in PM would reduce P-gp–mediated efflux at the cell membrane, thereby restoring drug sensitivity. To confirm this hypothesis, we loaded doxorubicin (DOX) into the PM (PM/D), and evaluated the release kinetics and cellular responses in MCF-7/ADR cells. In vitro, ultrasound markedly accelerated doxorubicin (DOX) release from PM/D, which resulted from polymer chain motion and network relaxation in nanogels induced by ultrasound. And ultrasound treatment increased intracellular DOX accumulation and cytotoxicity in MDR MCF-7/ADR cells. PM/D with ultrasound were able to decrease the IC50 from 37.89 ug/mL to 8.32 ug/mL, yielding a resistance reversion index of 4.55—approximately two-fold higher than PM/D without ultrasound. These results support a coherent mechanism whereby spatiotemporally triggered release elevates local drug activity to overcome the efflux threshold, and Pluronic-mediated efflux inhibition sustains intracellular retention. Therefore, we establish PM as a feasible and generalizable delivery platform for addressing MDR.

## 2. Results and Discussion

**Figure 2:**
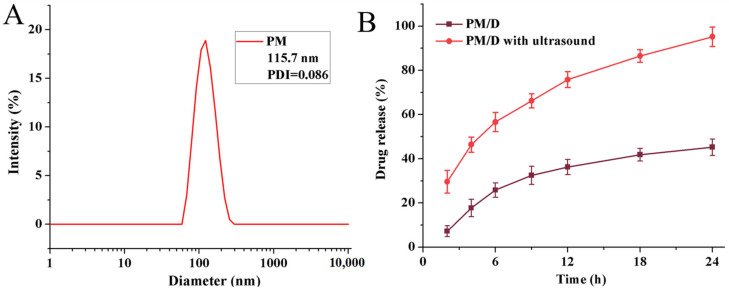
Average hydrodynamic diameter and distribution of PM (**A**); drug release performance of PM/D and PM/D after ultrasound (**B**). Mean ± SD of n = 5 independent samples.

Particle size is a primary determinant of biodistribution and tumor access. The particles in the 20~250 nm range often achieve a long circulation time in the blood and favorable retention in the tumor via the EPR effect. Thus, we firstly measured the diameter and size distribution of the Pluronic mixed nanogels (PM) with DLS. The corresponding results are shown in Figure 2A. The empty nanogels exhibited a low polydispersity index (PDI = 0.086), indicating a narrow size distribution. A mean hydrodynamic diameter of 115.7 nm was observed, which is conducive to accumulation at tumor sites through the EPR effect. The resulting hydrodynamic diameter falls squarely within the 100~150 nm range, which could be attributed to the complementary hydrophilic and hydrophobic properties between P105 and F127.

Doxorubicin was applied as a model drug to analyze ultrasonic responsiveness, and the corresponding results are shown in Figure 2B. The DOX-encapsulating efficiency of PM/D was 68.3%, indicating efficient drug loading into the hydrophobic cores. Additionally, controlled release was quantified in PBS with or without brief ultrasound. As shown in Figure 2B, PM/D exhibited minimal release in the absence of ultrasound. Only 7.2% DOX was released after 2 h of incubation. In sharp contrast, the ultrasound-treated group reached 29.6% DOX from PM/D nanogels. The release rate in the PM/D group with 3 min of ultrasound was approximately four times faster than that in the PM/D group without ultrasound. The result reflected the burst and on-demand release of DOX from the PM/D nanogels under the resistance of ultrasound. Moreover, over prolonged incubation, the divergence persisted. After 24 h of sustained release, 95% of loaded DOX could be released in the ultrasound-responsive group, compared with 45.2% in the control group. All phenomena might be caused by the chemical bond movement induced by ultrasonic effects. Under low-intensity ultrasound, stable cavitation and microstreaming loosen the network of nanogels for a short time. Thus, polymer chain exchange speeds up, which leads to the on-demand release of DOX. With longer or stronger pulses, the nanogels can break into smaller pieces, which further promotes drug release [39,40,41]. These data confirmed that PM nanogels are an ultrasound-responsive drug-delivery system that can controllably and efficiently release a payload using precise temporal control.

**Figure 3:**
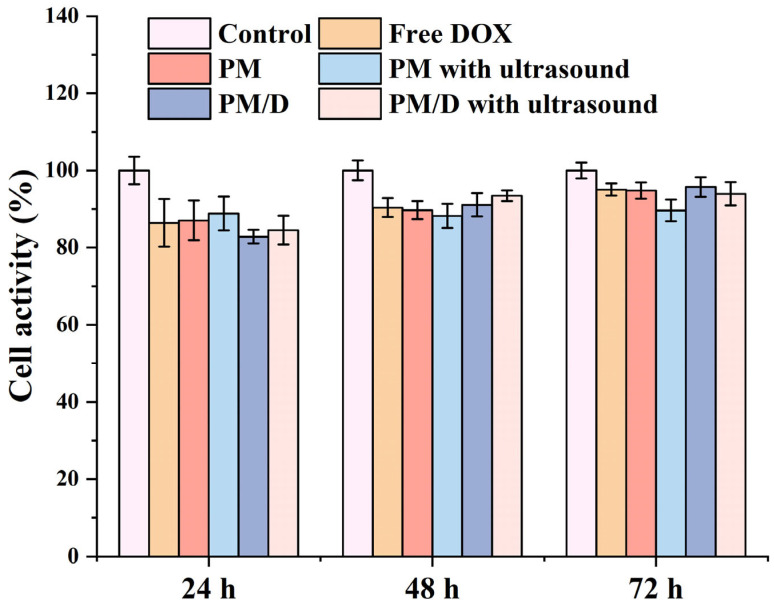
Cell viability of L929 cells after being treated with different treatments for 24, 48, and 72 h.

Prior to the antitumor study, the cytotoxicity of the PM/D nanogels was measured by determining L929 cell viability after incubation with various samples. As shown in Figure 3, cell viability was higher than 80% for all experimental groups. No significant difference in cell activity was observed in the experimental groups compared to the control group, which indicated high cytocompatibility of PM/D nanogels.

**Figure 4:**
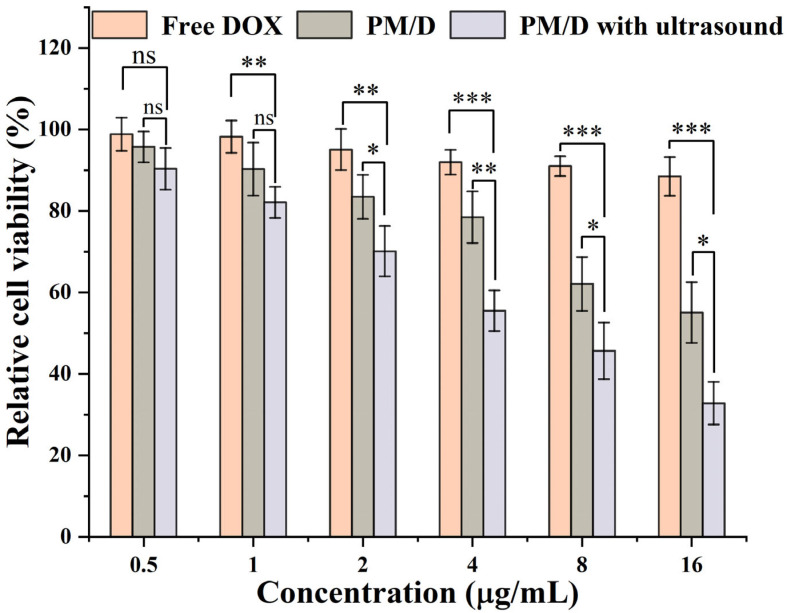
Toxicity assay of free DOX, PM/D and PM/D after ultrasound against MCF-7/ADR. Data are presented as the mean ± SD (n = 3). One-way analysis of variance (ANOVA) was used to test the validity. *p* < 0.05 is considered statistically significant. (ns: no significance, * *p* < 0.05, ** *p* < 0.01, and *** *p* < 0.001).

The MCF-7/ADR cell line was used for the investigation of the MDR reverse effect. The corresponding cytotoxicity analysis and MDR index are illustrated in Figure 4 and Table 1, respectively. As shown in Figure 4, DOX-resistance could be obviously observed in the free DOX group within the range of 0.5–16 µg/mL, with over 95% of cell viability treated with 16 μg/mL of DOX. The results suggested a typical MDR phenotype for MCF-7/ADR cells. Further application of ultrasound resulted in a decrease in cell viability and the most significant decrease at ≥4 µg/mL (reducing to approximately 50% of its original level compared to PM/D without ultrasound). Although dose-dependent cytotoxicity was observed in both the free and ultrasound-treated groups, a lower IC50 value was discovered with ultrasound-treated PM/D. In Table 1, the IC50 of free DOX was 37.89 µg/mL. The Pluronic mixed nanogels loading DOX (PM/D) reduced the IC50 to 16.54 µg/mL (RRI = 2.29, approximately 56% lower than the free drug), suggesting that the nanogel could partially alleviate the efflux mediated by P-gp and increase intracellular exposure. After applying ultrasound, the IC50 further decreased to 8.32 µg/mL. Likewise, the RRI in the ultrasound-treated PM/D group was 4.55 and was two times higher than that in the PM/D group without ultrasound, which represented better MDR reverse performance. These findings indicate that PM nanogels mediate MDR reversal through the combined effects of Pluronic-associated efflux modulation and ultrasound-triggered on-demand release.

**Figure 5:**
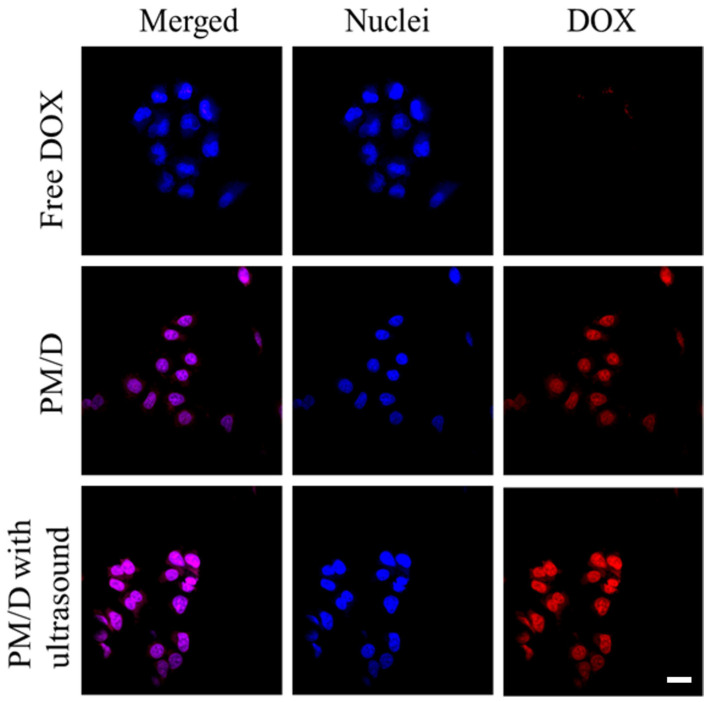
CLSM image of MCF-7/ADR cells after being treated with different DOX formulations for 6 h. The scale bar is 20 μm. Red channel, DOX; blue channel, DAPI.

**Figure 6:**
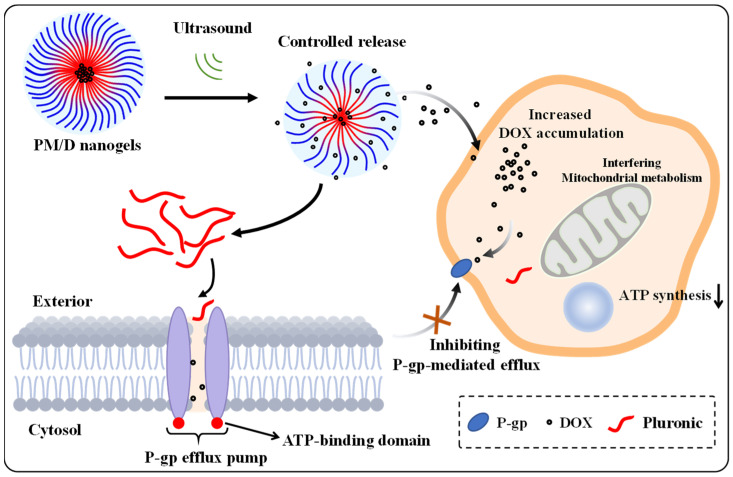
The mechanism of PM/D with ultrasound against MDR in cancer. Ultrasound-responsive PM nanogels enable on-demand DOX release. Low-intensity ultrasound induces transient network swelling and accelerates polymer chain exchange, triggering DOX release from the nanogels. Internalized Pluronic perturbs mitochondrial metabolism, reduces ATP synthesis, and functionally suppresses ATP-dependent P-gp efflux, resulting in increased intracellular DOX accumulation in tumor cells.

Additionally, we analyzed the cellular uptake and distribution of PM/D using LSCM after 6h of treatment. As shown in Figure 5, the red (DOX) fluorescence in the free DOX group was negligible, consistent with the powerful P-gp-mediated efflux in MCF-7/ADR cells. By contrast, the overall red signal in the PM/D group was significantly enhanced. The reason is that DOX-loaded nanogels homed into the cytoplasmic regions of MCF-7/ADR cells, and interfering with P-gp-mediated efflux induced by the Pluronic resulted in a high level of intracellular DOX content in Pluronic-involved groups. Moreover, after 3 min of ultrasound with PM/D, the strongest fluorescence intensity was observed compared to other groups, indicating the highest DOX concentration in MCF-7/ADR cells. This enhancement is consistent with ultrasound-triggered burst release increasing the local free DOX concentration at the membrane interface. Meanwhile, Pluronics can attenuate P-gp–mediated efflux by modulating membrane microviscosity, interfering with ATP synthesis and efflux activity, thereby restoring intracellular drug retention and reversing MDR in cancer cells. Thus, the contained Pluronic in PM/D could dampen P-gp-driven export to DOX, allowing intracellular DOX levels to rise above the efflux threshold required for nuclear engagement (Figure 6).

Compared with other ultrasound-sensitive nanocarriers, our system operates under low-intensity ultrasound, which causes only small temperature changes (ΔT ≤ 1–2 °C). Importantly, this low-intensity ultrasound does not fully disrupt the nanogels, which avoids the burst release of DOX and further maintains good biocompatibility. Moreover, combining ultrasound-responsive release with Pluronic-mediated efflux inhibition can significantly enhance the intracellular DOX accumulation in MCF-7/ADR cells. This gives a ~two-fold increase in RRI. Taken together, these results indicate that combining Pluronic-mediated efflux inhibition with ultrasound-controlled release can help reverse MDR in cancer models, offering an alternative strategy for treating MDR cancer.

## 3. Conclusions

In summary, self-assembled Pluronic P105/F127 nanogels provide an ultrasound-responsive platform to address multidrug resistance in cancer via a dual-action mechanism. Specifically, brief ultrasound had the functions of time control and deep penetration, capable of triggering local release, while Pluronic acted as a biochemical adjuvant, inhibiting P-gp-mediated efflux activity, thereby prolonging the retention time of intracellular drugs. The PM nanogels were prepared via simple mixing followed by spontaneous self-assembly, without chemical cross-linking and sophisticated fabrication. Thus, the PM system is readily reproducible, supporting clinical manufacturing. Moreover, the PM (~115.7 nm) passively accumulates in the tumor via the EPR effect, and ultrasound converts otherwise slow DOX leakage into on-demand release, boosting intracellular exposure and cytotoxicity in MCF-7/ADR cells. With ultrasound, cumulative release rose from 7.2% to 29.6% at 2 h, and the release rate increased over four-fold. In MCF-7/ADR cells, ultrasound-treated PM/D reduced cell viability by <56% at 4 µg/mL (vs. 78% of PM/D without ultrasound). The IC50 fell from 16.54 µg/mL to 8.32 µg/mL. Compared with the PM/D group without ultrasound, RRI in the ultrasound-treated PM/D group was 4.55, which represented better MDR reverse performance. These data position PM as a simple and externally controllable delivery system against MDR.

To advance toward clinical application, future work will need to consider the following: (i) Mechanism. RNA-seq and proteomics (Western blot of P-gp expression) can be used to analyze nanogel-induced reversal of resistance pathways (ABC transporters, apoptosis regulators). p (ii) In vivo validation. Antitumor activity against drug-resistant tumors should be evaluated in orthotopic drug-resistant mouse models. Device settings and patient factors may limit penetration and focus. Moreover, pharmacokinetic and biodistribution studies need to be conducted. (iii) Safety. The biocompatibility and repeat-dose toxicity of PM nanogels need to be assessed. Meanwhile, serum stability, protein corona, and clearance routes (hepatic/renal) should be defined. (iv) Indication. The PM can be extended to other chemotherapeutics or combined with sensitizers/P-gp modulators. Overall, we anticipate that the nanogels can offer a promising strategy to address MDR in cancer.

## 4. Materials and Methods

### 4.1. Materials

Pluronic P105 (PEG_37_PPG_56_PEG_37_; Mw 6500) and dimethyl sulfoxide (DMSO) were purchased from Aladdin Chemical Reagents Co., Ltd. (Shanghai, China). F127 (PEG_100_PPG_65_PEG_100_; Mw 12,600) was purchased from TCI (Shanghai, China). Phosphate buffer solution (PBS, 0.01M, pH 7.4) was purchased from Beijing Solarbio Science & Technology Co., Ltd. (Beijing, China). Doxorubicin hydrochloride (DOX) and cell counting kit-8 (CCK8) were provided by Sigma-Aldrich (St Louis, MO, USA) and KeyGEN BioTECH (Nanjing, China), respectively. (4′,6-diamidino-2-phenylindole) (DAPI) staining solution was purchased from ADAMAS-BETA Shanghai Luchi Trading Co., Ltd. (Beijing, China). Dialysis Tubing was obtained from Beyotime Science & Technology Co., Ltd. (Shanghai, China).

### 4.2. Preparation and Characterization of Pluronic Nanogels

#### 4.2.1. The Preparation of PM

The hydrophobic self-assembly method was used for the preparation of Pluronic mixed nanogels (PM). Briefly, 6.5 mg (0.001 mM) of P105 and 12.6 mg (0.001 mM) of F127 was dissolved in 2 mL of anhydrous DMSO. Then, the mixed solution was transferred to pre-soaked dialysis tubing (MWCO 3500) and dialyzed for 3 days with gentle stirring, replacing the external medium every 8–12 h to remove DMSO. Finally, the mixture was centrifuged twice at 12,000 rpm/min for 10 min and suspended with double-distilled water to discard unassembled free compounds. The obtained nanogels were stored at 4 °C until use. In addition, dynamic light scattering (DLS) was used to measure the hydrodynamic diameter and size distribution of the PM at room temperature.

#### 4.2.2. The Preparation of PM/D

The preparation of DOX-loading Pluronic mixed nanogels (PM/D) was similar to the above process. The 4 mg of DOX was added to the anhydrous DMSO solution in which P105 (6.5 mg) and F127 (12.6 mg) were dissolved. Then, the mixed solution was transferred to pre-soaked dialysis tubing (MWCO 3500) and dialyzed for 3 days with gentle stirring, replacing the external medium every 8–12 h to remove DMSO. Finally, the mixture was centrifuged twice at 12,000 rpm/min for 10 min and suspended with double-distilled water to discard unassembled free compounds. The obtained nanogels were stored at 4 °C until use.

#### 4.2.3. Measurement of DOX-Encapsulating Efficiency

The encapsulating efficiency of DOX in PM/D nanogels was measured by determining the contents of non-encapsulated DOX remaining in the supernatant after encapsulation. The absorption of supernatant at 481 nm was determined using SpectraMax M2e Molecular Devices. The encapsulating efficiency was calculated based on the calibration curve of DOX according to the following equation: Encapsulating efficiency = (content of DOX in feed − content of DOX in supernatant)/(content of DOX in feed).

#### 4.2.4. Determination of the Release Behavior of the Nanogels

First, 6 mL of specific DOX content of the PM/D nanogel suspension was divided into two parts (3 mL each) and transferred into a dialysis tube with 30 mL phosphate buffer solution (PBS, 0.01 M, pH 7.4) at 37 °C and 120 r/min under continuous stirring. In addition, the experimental group was immediately irradiated by ultrasound (20 kHz, 45 W, 3 min) while the temperature was held at 37 °C. The control received no ultrasound. At pre-set time intervals (2, 4, 6, 8, 12, 18, and 24 h), the 1 mL supernatant was withdrawn and replaced with an equal volume of fresh PBS. Then, the amount of released DOX was measured via UV-Vis spectrophotometry at 481 nm and analyzed using a standard curve. The cumulative release (%) was calculated. All experiments were performed in triplicate (n = 5) with identical acquisition settings.

#### 4.2.5. In Vitro Biocompatibility

The Cell Counting Kit-8 (CCK-8) was utilized to assess the nanogels’ safety in vitro. The assay uses the water-soluble tetrazolium salt WST-8 2-(2-methoxy-4-nitrophenyl)-3-(4-nitrophenyl)-5-(2,4-disulfophenyl)-2H-tetrazolium, monosodium salt to form a water-soluble formazan dye in cell culture medium. Cellular dehydrogenases reduce WST-8 to this dye only in living cells. The amount of produced dye is directly proportional to the number of viable cells, so a higher signal indicates more live cells. Briefly, approximately 5.0 × 104 L929 fibroblast cells per well were seeded into 96-well plates and cultured for 24 h. Then, the medium was replaced with fresh medium containing various samples. After 24, 48 h, and 72 h, 20 μL of CCK-8 solution was added to each well according to the manufacturer’s instructions. After 10 min of incubation, the absorbance was read at 450 nm with a microplate reader. Cell viability was calculated as follows: cell viability (%) = (OD sample/OD control) × 100%.

### 4.3. Cytotoxicity and Cell Uptake Test

#### 4.3.1. Cell Cytotoxicity

The CCK-8 assay was utilized to assess the PM nanogels against MCF-7/ADR human breast cancer cells (MCF-7/ADR). MCF-7/ADR is a DOX-resistant human breast cancer cell line generated through chronic DOX selection. The MCF-7/ADR cell line was obtained from Sunncell (Wuhan, China). Owing to the high expression of efflux transporter P-gp, it displays a typical multidrug resistance phenotype. P-gp-mediated efflux lowers intracellular DOX and drives MDR in cancer cells. Therefore, we employed MCF-7/ADR cells to evaluate whether the PM/D increases intracellular drug accumulation/killing efficacy in this experiment. DOX-resistant human breast cancer cells at 1 × 10^5^ cells/well were seeded into 96-well cell plates. After culturing for 24 h, the medium was removed. Then, the PM/D suspension with or without ultrasonic treatment at a gradient of drug concentrations was fed into the plate and further co-cultured for 24 h. The 20 μL of CCK8 solution was then added into each well for 2 h of incubation according to the corresponding manufacturer’s protocol. The absorbance of each well was measured using a microplate reader at 450 nm. Additionally, MCF-7/ADR cells treated with free DOX were set as the positive control group, MCF-7/ADR cells treated with saline were set as the negative control group, and 100% cell viability was achieved. Half-maximal inhibitory concentration (IC50) values are commonly used to quantify cellular drug sensitivity and provide insight into tumor resistance. Thus, we calculated IC50 to measure cellular sensitivity to the drug. Furthermore, we evaluated PM/D with ultrasound-enabled MDR reversal using the Resistance Reversion Index (RRI), defined by the following: RRI = IC50 of free DOX/IC50 of PM/D (+ ultrasound). RRI > 1 indicates resistance reversal.

#### 4.3.2. Cell Uptake Test

To study the cell uptake of PM/D, laser scanning confocal microscopy (LSCM) was utilized to visualize the DOX distribution after treatment with various formulations. Briefly, MCF-7/ADR cells (approximately 1.0 × 10^6^ cells per well) were seeded into 96-well plates. After culturing for 24 h, the medium was replaced with the PM/D suspension. Next, the cells were incubated for 6 h with the PM/D with or without ultrasound at 8 μg/mL. The MCF-7/ADR cells treated with free DOX were set as the control group. After incubation, all samples were washed twice with PBS and then stained with DAPI (containing antifade reagent, 5–10 min). Finally, all samples were scanned and photographed using LSCM (DAPI: Blue channel; DOX: Red channel).

## Figures and Tables

**Table 1:** The MDR index of various DOX formulations.

Formulation	IC 50 (μg/mL)	RRI ^a^
Free DOX	37.89	-
PM/D	16.54	2.29
PM/D with ultrasound	8.32	4.55

**^a^** Resistance reversion index (RRI), determined by the ratio of IC50 of free DOX to another group.

## Data Availability

The original contributions presented in the study are included in the article; further inquiries can be directed to the corresponding authors.
